# Mid-Second Trimester Measurement of Nasal Bone Length in North Indian Population

**DOI:** 10.25259/JCIS-15-2019

**Published:** 2019-05-24

**Authors:** Shreshtha Jain, Sachin Khanduri, Mazhar Khan, Shahla Khan, Vivek K. Yadav, Basmah R. Khan, Umar Faizan Sagar, Mridul Rajurkar

**Affiliations:** Department of Radiodiagnosis, Era’s Lucknow Medical College and Hospital, Lucknow, Uttar Pradesh, India.

**Keywords:** Fetal nasal bone, Ultrasound, Gestational age

## Abstract

**Objective:**

Our objective for this study was to establish a reference range of normal fetal nasal bone length (NBL) from 14 to 22 weeks in a North Indian population.

**Materials and Methods:**

Pregnant women with gestational age (GA) from 14 to 22 weeks undergoing ultrasonography with a single live fetus and no complications in the fetus or mother were selected for the study. The fetal nasal bone was measured in 2060 pregnant women from 2014 to 2018. The measurement was done by the double operator method; three measurements were taken for each woman when her fetus was in the midsagittal plane, and the nasal bone was located between a 45 and 135° angle to the ultrasound beam. We performed follow-up evaluations of all neonates.

**Results:**

The rate of growth of the fetal nasal bone during different weeks of gestation is described by an equation where NBL =0.365×GA+ 2.5885, with a fit estimate of R^2^ = 0.97, *P* < 0.001. The median NBL increased with GA from 2.9 mm at 14 weeks to 5.8 mm at 22 weeks in a linear relationship. Our results in the North Indian population are similar to those in the South Indian population and differ from those in Chinese and Japanese populations.

**Conclusions:**

The NBL in North Indian fetuses at 14–26 weeks of GA has a linear relationship to the week of gestation.

## INTRODUCTION

Nasal bone is an indispensible parameter in the second-trimester ultrasound. Nasal bone measurements vary according to race and ethnicity. Most of the criteria and normogram have been established according to race and origin. Till date, there have been no established criteria for nasal bone measurement in the second trimester in North Indian population which is 199 million.^[1]^

The absence or hypoplasia of the fetal nasal bone in children with Down syndrome can be detected by the first- and second-trimester sonography.^[2]^ A depressed nasal bone and short neck with increased uncial thickness are some common features found at the time of ultrasonography and after delivery in neonates.^[3]^ Healthy fetal nasal bone length (NBL) values have been established in Caucasian, African, American, and South American populations.^[4]^ Thus, ethnicity may impact fetal NBL. This study sought to determine a reference range for the second-trimester NBL in fetuses in a North Indian population and compare our findings with NBL findings reported in other populations.

## MATERIALS AND METHODS

This is a retrospective study conducted on North Indian pregnant women ranging from 14 to 22 weeks of gestation conducted in Era’s Lucknow Medical College and Hospital in Lucknow from 2014 to 2018. In our study, 2060 North Indian women who underwent ultrasonography were recruited. Informed consent was taken and ethics committee approval was obtained. All patients were registered under PC-PNDT Act of India, 1994. The criteria used for exclusion were abnormal karyotypes, fetal anomalies, fetal death *in utero*, absent nasal bone, and structural anomalies.

**Figure 1:**
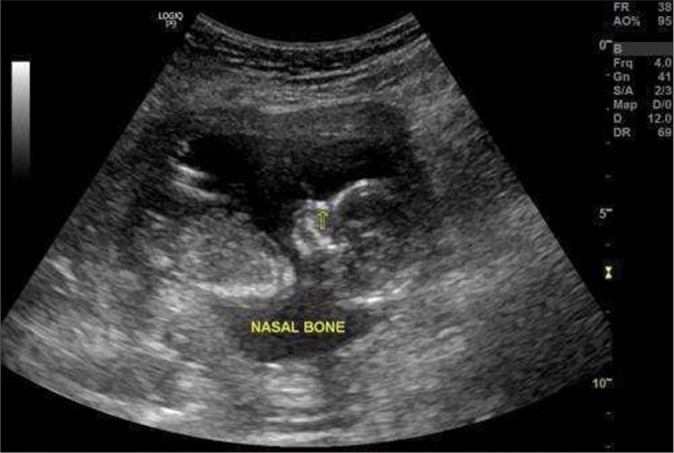
Sagittal gray scale ultrasound image of an intrauterine fetus of gestational age 16 weeks, 3 days showing the measurement method of fetal nasal bone (arrow) as described by Sonek *et al.*^[5]^

The measurement of the fetal NBL was performed by the double operator method with a mid-sagittal view of the fetal head to identify the nasal bone, lips, maxilla, and mandible with an angle from 45° to 135 degrees to the ultrasound beam, following the Sonek *et al.* method [Figure 1].^[5]^ Three images were obtained for each patient, and one measurement per image was taken for each patient. Fetal growth parameters (biparietal diameter, head circumference, abdominal circumference, and femoral length) were assessed for each fetus. In each case, fetal measurements were taken by ultrasound (Voluson P9, GE Health Care, Vienna, Austria). After data collection, statistical analysis was performed. The 5^th^ and 50^th^ percentile values of 2060 recruited pregnant women were calculated for the reference gestational weeks.

## RESULTS

**Figure 2:**
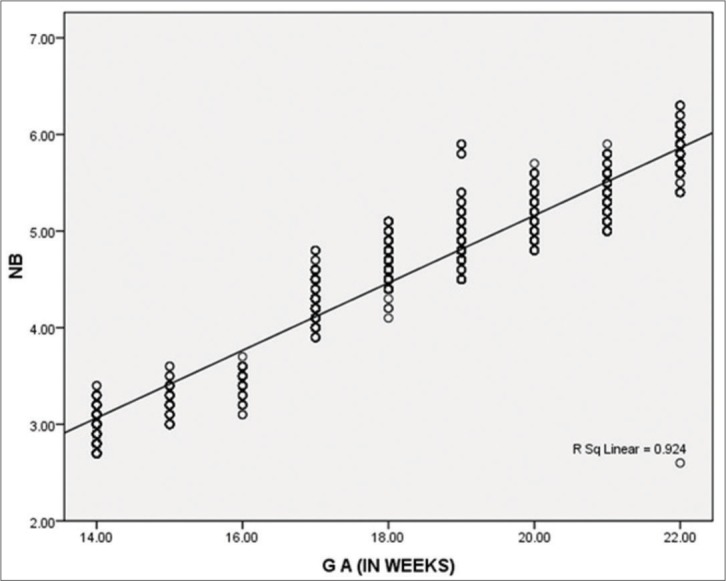
Scatter plot showing the relationship between mean fetal nasal bone length and gestational age of the fetus (14–22 weeks) in our study.

The rate of growth of the fetal nasal bone during different weeks of gestation is described by an equation where NBL = 0.365×gestational age (GA)+ 2.5885, with a fit estimate of R^2^ = 0.97, *P* < 0.001. The median NBL increased with GA from 2.9 mm at 14 weeks to 5.8 mm at 22 weeks in a linear relationship. The mean NBL was 4.63 mm, and the overall standard deviation was 0.925. The 5^th^ and 50^th^ percentile NBLs for each GA are shown in Table 1. The 5^th^ percentile signified the nasal hypoplasia/aplasia. The NBLs were closer to those of the South Indian populations, but our findings were different from those found in the Chinese and Japanese populations, as shown in Table 2.^[6-8]^ The difference in the mean was found to be statistically significant between North Indian population and Chinese population (*P* < 0.001). Mean and standard deviation values were unavailable for Japanese populations. The 50^th^ percentile of NBL in our North Indian population was also similar to that of South Indian populations, as shown in Table 3. The linear model was preferred for finding the relationship between mean NBL and GA as shown in the scatter plot of Figure 2.

**Table 1:** Percentile NBLs for each GA.

GA (weeks)	NBL (mm) by percentile						
	5^th^	10^th^	25^th^	50^th^	75^th^	90^th^	95^th^
14	2.700	2.700	2.800	2.900	3.000	3.200	3.200
15	3.000	3.000	3.100	3.200	3.300	3.400	3.500
16	3.155	3.200	3.300	3.500	3.600	3.600	3.600
17	4.000	4.000	4.100	4.200	4.400	4.500	4.600
18	4.400	4.500	4.500	4.700	4.800	4.900	5.000
19	4.600	4.700	4.800	4.900	5.100	5.200	5.300
20	4.900	4.900	5.000	5.100	5.200	5.300	5.400
21	5.100	5.100	5.200	5.300	5.500	5.600	5.600
22	5.400	5.600	5.700	5.800	5.925	6.100	6.100

GA: Gestational age, NBL: Nasal bone length

**Table 2:** NBL comparison with Japanese and Chinese populations.

GA (weeks)	50^th^ percentile NBL (mm)		
	Japanese population	Chinese population	North Indian population
	Kanagawa *et al.*^[7]^	Chen *et al.*^[6]^	Current study
14	ND	ND	2.9
15	3.2	3.5	3.2
16	3.5	4.1	3.5
17	4.5	4.6	4.2
18	4.9	5	4.7
19	5.2	5.6	4.9
20	5.8	5.8	5.1
21	5.7	6.2	5.3
22	6.6	6.7	5.8

GA: Gestational age, NBL: Nasal bone length, ND: Not determined

**Table 3:** NBL comparison with South Indian populations.

GA (weeks)	50^th^ percentile NBL (mm)	
	South Indian population	North Indian population
	Narayani and Radhakrishnan^[6]^	Current study
14	ND	2.9
15	ND	3.2
16	3.3	3.5
17	3.7	4.2
18	4.2	4.5
19	4.6	4.8
20	4.9	5.0
21	5.3	5.2
22	5.7	5.8

GA: Gestational age, NBL: Nasal bone length, ND: Not determined

## DISCUSSION

Association of hypoplastic or absent nasal bone with various congenital anomalies makes it an indispensable parameter of second-trimester ultrasound. Nasal bone measurements vary according to race and ethnicity.

In contrast to Narayani and Radhakrishnan, the NBL at 16 and 22 weeks in our study was 3.5 mm and 5.8 mm, respectively, which was on the higher side as compared to them.

In the previous studies, the isolated finding of absent/hypoplastic nasal bone was associated with chromosomal disease in 10%–12% of cases. Ultrasound anomalies were noted in 41.4% of absent/hypoplastic nasal bone cases, and abnormal chromosomes were noted in 28.5% of cases involving high-risk biochemical studies.^[3]^ These detection rates were likely similar to those of the second-trimester maternal serum biochemical screening tests.^[8]^

## CONCLUSIONS

NBL is an objective measurement in clinical practice. The NBL in North Indian fetuses at 14 to 26 weeks of GA has a linear relationship to the week of gestation. Our study confirmed significant differences in the 50^th^ percentile NBLs between Chinese and Japanese populations and our north Indian population, and we found a close relationship with subtle differences between North and South Indian populations.
